# Is Repeated Hepatic Resection Justified for Malignancy?

**DOI:** 10.1155/1991/82079

**Published:** 1991

**Authors:** Paul H. Sugarbaker

**Affiliations:** The Cancer Institute Washington Hospital Center 110 Irving Street, NW Washington DC 20010-2975 USA


### IS REPEATED HEPATIC RESECTION JUSTIFIED FOR MALIGNANCY?

### ABSTRACT

*Huguet, C., Bona, S., Nordlinger, B., Lagrange, L., Parc, R., Harb, J. and Benard, F.. (1990) Repeat hepatic resection for primary and metastatic carcinoma of the liver. Surgery, Gynecology & Obstetrics:* **171**: 398-402

**During the last 15 years, 19 patients underwent repeated hepatic resections for malignant lesions of the liver. The first hepatic resection had been performed four to 40 months earlier for treatment of hepatocellular carcinoma (nine patients) or hepatic metastases (ten patients), eight of which were of colorectal origin. Repeat resection was an extensive hepatectomy in six, a segmentectomy in six and a local excision in seven. In one patient, three wedge resections and, finally, hepatic transplantation were subsequently performed after an initial extended right lobectomy. The operative mortality rate was 5.2 per cent. The three year actuarial survival rate was 64 per cent after the second resection.**

### PAPER DISCUSSION

KEY WORDS: Liver resection, hepatocellular carcinoma, colorectal hepatic metastases

Huguet and his colleagues present yet further evidence that liver surgery for primary or metastatic cancer is not a final surgical treatment modality for patients with metastatic or primary liver tumors. This manuscript tells us that beneficial results from repeat resection of these tumors is possible. At a time when many physicians barely accept that surgery is a treatment alternative for this group of patients, this paper may have some difficulty in receiving a broad implementation. Yet, this paper is part of a growing literature supporting the practice of repeat hepatic resections. This paper as others, finds surprisingly good results of surgical treatment in this very select patient population[1-11].

### *Tumor Biology of Repeat Resection of Hepatic Tumors*

From a tumor biology perspective, repeat resections of primary and metastatic cancer to the liver finds a firm basis. It's well substantiated that hepatocellular carcinoma may occur as a focal process within the liver. The same carcinogenic events which lead to the first primary tumor can result in a second or even third disease focus. In other patients, of course, limited margins of resections combined with intrahepatic dissemination of cancer through the generous lymphatic channels of the liver may result in a local recurrence. The wedge excision of a primary liver tumor or even trisectorectomy as a repeat hepatic resection may result in the surgical removal of a second primary tumor or marginal recurrence and therefore long term disease free survival.

For colorectal metastases to the liver the biologic rationale for this procedure is sometimes the same but usually quite different. Occasionally, local recurrences within the liver parenchyma may result. With modern day radiology and liver parenchyma dissection technology this unfortunate occurrence should be a thing of the past.

In the majority of patients with colorectal metastases to the liver (or other tumors giving off tumor emboli to the portal system) the clinician sees over time the clinical presentation of occult disease in the liver. All liver resectionists at this point in time accept the fact that the results of treatment of this disease vary directly as ones ability to detect occult foci of tumor both within the liver and at other systemic sites. Of course, the CT scanner in the detection of liver, lung and retroperitoneal disease has gone a long way to improve the results of treatment. As foci of occult disease grow out over time the astute clinician will attempt to use repeat resections to render the patient clinically disease free and capable of long term disease free survival.

The concept of metastatic inefficiency is extremely important in understanding the tumor biology rationale in patients with repeat hepatic resections[12]. Metastatic inefficiency interprets implantation as an unusual event for tumor emboli present within "endothelial lined basement membrane intact vascular or lymphatic channels". Interpreted in an evolutionary perspective this body compartment was designed to transport white cells, red cells, and platelets. Tumor cells enter at their own risk. Billions of tumor cells are destroyed by a natural process for one tumor cell to adhere, implant, grow, persist, and progress as a focus of metastatic disease.

Some basic science investigators have suggested that some tumors may only have the biological equipment to implant at a single site[13]. The integrin system may confer an organ specific mechanism of adherences. Even though some tumor cells lines may be injected systemically, the metastatic process turns out to be a selective process within liver parenchyma. Only liver metastases develop because of unique liver specific cell surface receptors on the cancer cells that gain access to the blood stream.

The phenomenon that requires repeat resection of primary and metastatic liver tumors occurs only in a small proportion of the total patient population. Hepatocellular carcinoma is an unusually agressive disease process. Recurrences within the liver are seen to involve major portal venous or major hepatic venous structures. Repeat resection is usually not compatible with sufficient viable liver parenchyma for patient survival following the surgery. It's the unusual patient that can undergo a repeat resection[14]. A low grade of tumor biology as demonstrated by well differentiated or fibrolamallar histology is recorded in a majority of patients with repeat resections of primary liver tumors. Of course, limited or absent liver regeneration in patients with cirrhosis further reduces the number of candidates for repeat resection.

Numerous studies regarding the patterns of surgical treatment failure following liver resections for metastatic colorectal cancer have appeared in the literature[15-18]. As emphasized by these authors, systemic recurrence or combinations of systemic recurrence plus liver recurrence are seen in the great majority of patients with recurrent disease following liver resection. Studies reported by Griffith and colleagues suggested that one in ten liver resection patients may require a repeat resection[6].

*Survival Benefits as a Result of Surgical Complete Response*

Although Huguet and colleagues call our attention to repeat liver metastases, one should not forget that disease dissemination to other sites in this patient population is frequently seen. Synchronous or metachronous lung metastases in patients with large bowel cancer should be considered for surgical removal[19]. Long term survivals after liver resection plus lung resection do occur. If one can achieve a complete response (CR) by surgery the longevity of the patient is prolonged. If these CR's can be achieved with minimal morbidity and mortality and are compatible with a good quality of life following surgery for metastatic disease, then one can with confidence continue to prolong survival by inducing a surgical CR through the resection of metastatic cancer.

This concept is not as well supported in the literature for hepatoma as it is for colorectal metastases. Large bowel cancer patients made clinically disease free of liver metastases, lung metastases and suture line or resection site recurrence usually profit with prolonged survival. Recent clinical studies from our group suggest that some patients with limited peritoneal seeding who have complete resection plus intraperitoneal chemotherapy may survive long term[20]. More follow up is needed in these patients with peritoneal carcinomatosis. However, in summary it's safe to say that reoperative surgery that induces in the patient a complete response results in a prolongation of survival. Five year disease free survival will occur in a lesser group of patients.

*Chemotherapy to Delay the Appearance or Decrease the Incidence of Systemic Disease*

Recent studies in the United States suggest that combination chemotherapy may be successful in reducing systemic disease and improving long term survival in patients with Duke's C colon cancer[21]. This patient population is quite similar to patients who have liver resection for metastatic colorectal cancer. Although controlled studies are not currently available, one suspects that aggressive chemotherapy employed early on after liver resection may result in substantial improvements in survival of patients with resected hepatic metastases. The studies of Hodgson, Friedland, and Ahmed,[22] *et al.*, and Kemeny, Goldberg, and Beatty, *et al*,[6] suggest that the clinical evidence of recurrent hepatic metastases may be diminished by intraarterial chemotherapy. Further studies with combinations of surgical and chemical liver cytoreduction are underway.

The mortality of the repeat hepatic resections seems reasonable but it is different from liver resections for cancer the first time around. Ten of nineteen patients in this series developed a major complication and two patients had two different postoperative complications. This emphasizes the need to develop prognostic indicators in this group of patients. The influence of free interval, multiplicity, preoperative tumor antigen assays and tumor histology need to be determined for this patient population. Especially the margin of resection of the first and second liver resection should be correlated with survival.

*Medical Economics of Liver Resection*

In an era of increasing austerity in health care the Huguet, *et al.* manuscript causes us to take notice. Our authors suggest that a proportion of patients who fail one liver resection can benefit from a second. Meticulous follow up is indeed expensive. Follow up requires frequent visits to a physician, serial tumor antigen assays and radiologic studies. Perhaps, at least in some part, the success in these patient populations depends on the successful use of tumor markers. AFP is a marker for hepatocellular cancer and CEA, or CA19-9 are markers for colorectal cancers. A large proportion of patients with liver metastases will have progressively elevating CEA titers. One suspects that the same is true for recurring hepatoma although the clinical research supporting this is not readily available. Liver radiology using ultrasound in Europe or CT scanning in the United States has improved not only technically but in its wider application. With these follow up patients can be detected at a resectable stage of disease. By Huguet and coworkers data, these patients are at risk for recurrent liver tumors that can be resected for up to three years. We have seen recurrent liver tumors up to six years following the first liver resection (unpublished data).

We recommend the same follow up schedule for patients with liver metastases as we do with those with Duke's B or C colorectal cancer[23]. The 2,4,6 plan is employed in order to diagnose early recurrences. CEA assays and a visit to a healthcare professional is recommended every 2 months for the first postoperative year. In the second and third postoperative year we see the patients every 4 months. And, in the fourth and fifth years every 6 months. A chest radiograph and a liver imaging study (either ultrasound or CT) is recommended every six months for 3 years and then on a yearly basis. Clearly, this approach will lead to an escalation in the cost of care of these patients but must be recommended if one is to optimize the care of the patients with large bowel primary and metastatic liver tumors.

### REFERENCES

1. Shepard, K.V., Levin, B., Karl, R.C., *et al.* (1985) Therapy for metastatic colorectal cancer with hepatic artery infusion chemotherapy using a subcutaneous implanted pump. *J.Clin.Oncol.*, **3**, 161
2. Cohen, A.M., Kaufman, S.D., Wood, W.C., *et al.* (1983) Regional hepatic chemotherapy using an implantable drug infusion pump. *Am.J.Surg.*, **145**, 529–533
3. Weiss, G.R., Garnick, M.B., Osteen, R., *et al.* (1983) Long-term hepatic arterial infusion of 5-fluorodeoxyuridine for liver metastases using an implantable infusion pump. *J.Clin.Oncol.*, **1**, 337–344
4. Schwartz, S.I., Jones, L.S. and McCune, C.S. (1985) Assessment of treatment of intrahepatic malignancies using chemotherapy via an implantable pump. *Ann.Surg.*, **201**, 560–567
5. Johnson, L.P., Wasserman, P.B. and Rivkin, S.A. (1983) FUDR hepatic arterial infusion via an implantable pump for treatment of hepatic tumor. *Proc.Am.Soc.Clin.Oncol.*, **2**, 119
6. Kemeny, M.M., Goldberg, D., Beatty, J.D., *et al.* (1986) Results of a prospective randomized trial of continuous regional chemotherapy and hepatic resection as treatment of hepatic metastases from colorectal primaries. *Cancer*, **57**, 492
7. Ramming, K.P. and O'Toole, K. (1986) The use of the implantable chemo-infusion pump in the treatment of hepatic metastases of colorectal cancer. *Arch.Surg.*, **121**, 1440–1444
8. Kemeny, N., Daly, J., Reichman, B., *et al.* (1987) Intrahepatic or systemic infusion of fluorodeoxyuridine in patients with liver metastases from colorectal carcinoma. *Ann.Intern.Med.*, **107**, 459–465
9. Hohn, D., Stagg, R., Friedman, M., *et al.* (1987) The NCOG randomized trial of intravenous (IV) vs hepatic arterial (IA) FUDR for colorectal cancer metastatic to the liver. *Proc.ASCO*, **6**, 65
10. Chang, A.E., Schneider, P.D. and Sugarbaker, P.H. (1987) A prospective randomized trial or regional versus systemic continuous 5-fluorodeoxyuridine chemotherapy in the treatment of colorectal liver metastases. *Ann.Surg.*, **206**, 685–693

11. Niederhuber, J.E. (1985) Arterial chemotherapy for metastatic colorectal cancer in the liver. Conference Advantages in Regional Cancer Therapy. Giessen, West Germany
12. Weiss, L. (1989) Metastatic inefficiency and regional therapy for liver metastases from colorectal carcinoma. *Reg.Cancer Treat.*, **2**, 77–81
13. Albelda, S.M. and Buck, C.A. (1988) Integrins and other cell adhesion molecules. *FASEB J*, **4**, 2868–2880
14. Kanematsu, T., Matsumata, T., Takenaka, K., *et al.* (1988) Clinical management of recurrent hepatocellular carcinoma after primary resection. *Br.J.Surg.*, **75**, 203–206
15. Hughes, K.S., Sugarbaker, P.H. and other members of the Hepatic Metastases Registry. (1986) Resection of the liver for colorectal carcinoma metastases: A multi-institutional study of patterns of recurrence. *Surgery*, **100**, 278–284
16. Steel, G. Jr., Osteen, R.T., Wilson, R.E., Brooks, D.C., Mayer, R.J., Zamcheck, N. and Ravikumar, T.S. (1984) Patterns of failure after surgical cure of liver tumors. A change in the proximate cause of death and a need for effective systemic adjuvant therapy. *Am.J.Surg.*, **147**, 554–559
17. Bozzetti, F., Doci, R., Bignami, P. and Genarri, L. (1987) Patterns of failure following surgical resection of colorectal cancer liver metastases: rationale for a multimodal approach. *Ann.Surg.*, **205**, 264–270
18. Ekberg, J., Tranberg, K.G., Andersson, R., *et al.* (1987) Pattern of recurrence in liver resection for colorectal secondaries. *World J.Surg.*, **11**, 541–547
19. Kern, K.A., Pass, H.I. and Roth, J.A. (1987) Surgical treatment of pulmonary metastases. (In) Rosenberg SA. *Surgical Treatment of Metastatic Cancer*, pp 69–100. J.B.Lippincott Company: Philadelphia
20. Sugarbaker, P.H. (1991) Cytoreductive approach to peritoneal carcinomatosis: peritonectomy and intraperitoneal chemotherapy. *Post Advances in Colorectal Surgery*, **11-X**, 1–14
21. Consensus Statement. (1990) Adjuvant therapy for patients with colon and rectum cancer. NIH Consensus Development Conference, April 16–18, 1990, Vol.8 No.4
22. Hodgson, W.J.B., Friedman, M., Ahmed, T., *et al.* (1986) Treatment of colorectal hepatic metastases by intrahepatic chemotherapy alone or as an adjuvant to complete or partial removal of metastatic disease. *Ann.Surg.*, **203**, 420–425
23. Sugarbaker, P.H., Gianole, F.J., Dwyer, A.J. and Neuman, R. (1987) A simplified plan for follow-up of patients with colon and rectal cancer supported by prospective studies of laboratory and radiologic test results. *Surgery*, **102**, 79–83

Paul H Sugarbaker
Medical Director
The Cancer Institute
Washington Hospital Center
110 Irving Street, NW
Washington DC 20010-2975
United States of America

### THE WARREN SHUNT: EFFECT OF ALCOHOLISM ON PORTAL PERFUSION

### ABSTRACT

*Kawasaki, S., Henderson, J.M., Hertzler, G. and Galloway, J.R. (1991) The role of continued drinking in loss of portal perfusion after distal splenorenal shunt. Gastroenterology;* **100**, 799-804

